# Periodontitis in First Degree-Relatives of Individuals With Rheumatoid Arthritis: A Short Narrative Review

**DOI:** 10.3389/froh.2022.895753

**Published:** 2022-05-06

**Authors:** Alkisti Zekeridou, Benoit Gilbert, Axel Finckh, Catherine Giannopoulou

**Affiliations:** ^1^Division of Regenerative Dental Medicine and Periodontology, University Clinics of Dental Medicine, University of Geneva, Geneva, Switzerland; ^2^Division of Rheumatology, Geneva University Hospitals (HUG), Geneva, Switzerland

**Keywords:** first degree relatives, rheumatoid arthritis, periodontitis, ACPA, oral microbiota

## Abstract

Periodontal disease (PD) and rheumatoid arthritis (RA) are chronic inflammatory diseases with a bi-directional relationship. Both share common genetic and environmental risk factors and result in the progressive destruction of bone and connective tissue. First degree relatives of patients with RA (FDR-RA) are one of the at-risk populations for RA. The etiopathogenic mechanisms of their susceptibility are currently being explored, focusing mostly on the role of anti–cyclic citrullinated protein/ peptide antibodies (ACPA) in triggering RA. Oral microbiota and their relation with oral health has been suggested as a factor influencing the risk of the FDR-RA developing RA. In particular, compromised periodontal status often correlates with ACPA seropositivity in FDR-RA. The presence of periodontal pathogens such as *Porphyromonas gingivalis*, in oral microbiota has been proposed to increase the risk of developing RA through its uniquely expressed peptidyl arginine deiminase (PPAD), capable of citrullinating both host and bacterial peptides. *Aggregatibacter actinomycetemcomitans* and its leukotoxin A (LtxA), also induces hypercitrullination in host neutrophils. Common risk factors of periodontitis and RA such as genetic predisposition, smoking, higher local and systemic inflammatory burden, are discussed in the literature. Based on those mechanisms periodontal disease seems to be presented as one of the factors triggering RA in FDR-RA. Larger studies evaluating all the potential mechanisms linking RA and periodontitis are needed in FDR-RA to confirm that periodontal disease should be considered in the screening of FDR-RA.

## Introduction

Before the clinical onset of rheumatoid arthritis (RA), a preclinical period exists during which genetic and environmental factors interact to initiate the autoimmune process. The ensuing autoimmune response is characterized by the production of rheumatoid factor (RF) and/or anti-citrullinated protein antibodies (ACPA). According to EULAR Standing Committee, this preclinical phase could be divided in three ≪at risk≫ stages: genetic and environmental risk, including first-degree relatives (FDR-RA) of patients with RA, systemic autoimmunity associated with RA and symptomatic preclinical phases [1].

This review focuses on FDR-RA, as defined by the EULAR terminology, who are good candidates for clinical and biomarker profiling, providing insight in the etiology of RA [2]. Our aim is to discuss the available evidence on mechanisms linking periodontitis and RA onset in this population.

### RA and First-Degree Relatives

FDR- RA have a 3 to 5-fold increased risk of developing the disease [3].While the shared epitope (SE) is the genetic factor which has been most associated with RA, genome wide association have revealed dozens of RA-associated single-nucleotide polymorphisms [4]. Based on twin studies, RA has been estimated to have an overall 30-60% heritability [5], predominantly for seropositive RA [6], with ~30–40% owing only to SE [7, 8]. Still, researchers have suggested that genetic predisposition might lead to RA only when encountering certain environmental conditions. Such gene-environment interaction has been demonstrated between the SE-positive human leukocyte antigen (HLA) alleles and inhaled pollutants, such as tobacco smoking, in seropositive RA patients [9–11]. The combination of smoking and double shared epitope increased the risk of RA up to 21-fold [95% CI (11–40)] [9]. It has to be underlined that SE positivity associates essentially with ACPA-positive RA and increases the risk of cardiovascular mortality [12]; suggesting subgroup-specific pathogenic mechanisms [13].

### RA and Periodontal Disease

Mucosal exposure to exogenous antigens impacts the immune system [14]. Studies in healthy individuals demonstrated that IgA antibodies, such as ACPA and RF, can be secreted at mucosal sites in response to local inflammation [15, 16]. These auto-antibodies are frequently found in the sputum of RA patients, even when they are undetectable in the serum [17]. The latter suggests that the development of systemic auto-immunity could be a consequence of chronic mucosal barrier disruption, local immune activation, and subsequent systemic spread of auto-reactive cells. This theory is known as the “mucosal origins hypothesis” [15].

Chronic intestinal conditions [18], or chronic pulmonary disorders [19, 20], have been linked with RA. Similarly, periodontal disease (PD) [21–23] has been proposed as a trigger for RA. Periodontitis is mediated by an interplay between dysbiotic microbial communities and aberrant host immune-inflammatory responses within the gingival and periodontal tissues. The dysbiotic plaque biofilm contains high proportions of Gram-negative, anaerobic and facultative bacteria, and is microbially less diverse than a healthy biofilm. The term “keystone pathogen” is used to describe bacteria that support and stabilize a microbiota associated with disease and cause disruption of host-microbial homeostasis [24]. *Porphyromonas gingivalis* (P. *gingivalis*) is a keystone pathogen strongly associated with periodontitis by directly affecting the resident oral microbiota and indirectly modulating the native immune system. Individuals' susceptibility of developing periodontitis can also be affected by genetics, epigenetic factors and environmental lifestyle factors, such as suboptimal oral hygiene, stress, smoking, systemic conditions, medication, diet any many others [25].

A bi-directional relationship between PD and RA has been revealed in cross-sectional studies. A higher prevalence of periodontitis has been reported in patients with RA compared to healthy controls with odds-ratios (OR) ranging from 1.82 to 8.05 after adjusting for confounding factors such as plaque accumulation and gingival inflammation [26–28]. Conversely, an increased prevalence of RA has been found in patients with periodontitis, compared to periodontally healthy subjects (OR ranging from 1.16 to 4.28) [22, 23, 29]. Furthermore, the severity of periodontitis correlates with RA disease activity [30]. However, some studies failed to show significant differences in periodontitis prevalence between RA subjects and non-RA subjects [31]. The contradictory results may be attributed to different adjustments for confounding variables between studies (comorbidities, RA activity, medication) and differences in disease classification criteria for periodontitis.

## Link Mechanisms Between RA and PD

Periodontitis is included in the “two-hit” model for RA etiology, introduced by Golub et al. [32]. The first hit represents ACPA production due to chronic periodontitis followed by a second hit in the joint that induces RA. In other words, abnormal and bacterial citrullination by *P. gingivalis* within the periodontal tissue results first in a local autoimmune response to citrullinated proteins followed by the systemic production of ACPA in the joints that can induce RA [32].

ACPAs are the most specific antibodies associated with RA [33]. They are found in approximately 80% of the RA patients [34]. Their presence is highly associated with the HLA-shared epitope (SE), which is linked to the risk of developing RA and in particular for ACPA-positive RA. Interestingly, ACPA can appear years before the onset of RA, thus being a strong predictor of the disease [35].

Recent research has focused on the identification of external factors that could trigger such autoantibody production. The autoantibodies are produced following the excess formation of citrullinated proteins [36]. Citrullination refers to the post-translational process of the modification of the amino acid arginine into citrulline. The process is mediated by the peptidyle arginine deimase enzyme (PAD) of various immune cells such as the neutrophils, macrophages, monocytes and T and B lymphocytes. Five PAD enzymes have been identified in humans [37]; two isoforms of the PAD family, the PAD2 and PAD4 are expressed in inflamed periodontal tissues [38]. In addition to the human PADs, the periodontal pathogen P. *gingivalis*, has been shown to express a PAD enzyme (referred to as PPAD to distinguish from the human PAD) capable of citrullinating host and bacterial peptides. In particular, citrullinated fibrinogen and citrullinated alpha-enolase are targeted by anti-citrullinated protein antibodies. These autoantibodies are found, respectively, in up to 60% and 40-60% of patients with RA [39].Thus, citrullination associated with the host-derived PAD is further increased by the bacterial-derived PADs, leading to an enhanced production of ACPA [40].

In addition to its ability to express PPAD, P. *gingivalis* can induce the production of various pro-inflammatory cytokines by the immune cells, stimulate a Th17 response and accelerate the development of RA. It has been established that Th17 cell-related cytokines are strong inducers of arthritis and that IL-17 plays important role in the osteoclast differentiation and bone erosions [41, 42].

*A. actinomycetemcomitans*, another important periodontal pathogen, has been also proposed as a potential trigger for the pathogenesis of RA. The bacteria possess a virulence factor, a pore-forming leukotoxin A (LtxA) which can dysregulate the activation of citrullinated enzymes and induce hypercitrullination in host neutrophils [43].

Aside from formation of ACPA due to citrullination, other molecular pathways have been also studied to link PD with RA. First, uncontrolled generation of neutrophil extracellular traps (NETs) has been found in several autoimmune diseases in response to periodontal pathogens. Accumulated NETs provide a source of autoantigens in both PD and RA [44]. A second mechanism, described as molecular mimicry, involves the capacity of P. *gingivalis* and some other bacteria in dental plaque to express antigens, that are structurally similar to host antigens, and can therefore cross-react with ACPAs. Bacterial enolase and bacterial heat shock protein 60 are the strongest and mostly studied candidates that trigger an immune response and generate antibodies [45]. Other potential mechanisms linking PD to RA focused on the capacity of P. *gingivalis*, as shown in an experimentally-induced periodontitis animal model, to modulate the gut microbiota composition [46] and to be hematogenously disseminated to synovial joints [47].

Genetic and environmental risk factors are common between PD and RA resulting in the progressive destruction of bone and connective tissue [48]. The shared epitope (SE) coding HLA-DRB alleles are potential genetic elements connecting RA and PD [49]; they have been associated with bone erosions in RA and alveolar bone destruction and PD progression [50]. Moreover, family transmission of putative periodontal pathogens between family members has been documented [51].

Finally, tobacco consumption is an established risk factor for periodontal destruction and RA. Case-control studies have shown that in smokers, the risk of developing seropositive RA was twice higher compared to non-smokers and the risk was dose-dependent on lifetime exposure to smoking [52]. Likewise, smoking affects in a dose dependent way all aspects of periodontal health such as prevalence of PD, severity of periodontal destruction and unfavorable results to periodontal treatment [53]. Exogenic risk factors such as nutrition, socioeconomic status, psychological factors (stress) and obesity are common between the two pathologies [44].

## Clinical Evidence of The Relationship Between RA and PD

As mentioned above, periodontopathic microorganisms may trigger, deteriorate and perpetuate RA [54]. Citrullinated proteins have been detected in periodontal tissues and in inflammatory exudates [38, 55]. Thus, PD could act as an environmental stressor for ACPA-positivity. The hypothesis is that in genetically susceptible individuals, citrullination associated with periodontitis may cause a localized oral mucosal response, which can lead to a systemic ACPA production and the onset of RA.

The clinical evidence for the relation of PD and RA is large and variant. The Nagahama study included 9,575 subjects with no connective tissue disease and showed significant associations between periodontal parameters and ACPA seropositivity. In this population 27.9% were ACPA-positive, supporting the involvement of PD in ACPA production [56]. However, when serum levels were analyzed for ACPA quantification in subjects with or without RA, and with or without PD, no correlation was found between ACPA and the clinical parameters of PD [57].

Conflicting data and opinions have been reported regarding the relationship between periopathogenic bacteria and RA. In some studies, the subgingival presence of P. *gingivalis* and A. *actinomycetemcomitans* and the levels of serum anti- *Porphyromonas gingivalis* and anti- *Aggregatibacter actinomycetemcomitans* immunoglobulins were not associated with RA [58]. While other publications reported a weak but significant correlation between anti- *Porphyromonas gingivalis* outer membrane levels and ACPA titers [59]. The study of Schmickler et al. [60], revealed a higher number of *Fusobacterium nucleatum* and P. *gingivalis* in ACPA seropositive patients with RA. In cases of untreated new-onset RA, P. *gingivalis* was identified in 55% of the patients [60] whereas in the study of Bello-Gualtero et al. [61], P. *gingivalis* specific IgG was found to be associated with ACPA in early-RA, but not in pre-RA. This supports the hypothesis that P. *gingivalis* infection plays a role in the early loss of tolerance to potential self-antigens during the RA pathogenesis [61]. The study of Fisher et al. [62] agreed that P. *gingivalis* was not associated with pre-RA autoimmunity or risk of RA in an early phase before the disease onset.

## The FDR-RA and Periodontitis

All the above mechanisms linking PD and RA may potentially also apply to the susceptibility of FDR-RA of developing RA [2, 63]. However, only a limited number of studies evaluated the prevalence or indicators of periodontitis in FDR-RA [36, 64, 65]. These studies evaluated mainly the role of ACPA and oral microbiota, as main mechanisms.

### Clinical Evidence of the FDR-RA and Periodontitis

Focusing on the FDR-RA population, the study of Barra et al. [66], including 88 RA patients, 50 unaffected FDR-RA and 20 healthy control subjects, investigated ACPA along with self-reported joint and PD symptoms. FDR-RA had four times higher prevalence of ACPA compared to controls. Joint and PD symptoms in the FDR-RA were significantly associated with smoking. However, in this study the periodontal status was similar in the three groups. The small sample size and the similar hygiene attitudes between the groups may have overruled the potential differences [66].

On the other hand, Unriza-Puin et al. [67] found a difference in the periodontal status of FDR-RA compared to healthy participants. They investigated the body mass index (BMI), ACPA, the presence of periodontitis and the presence of IgG-1/ IgG-2 antibodies against P. *gingivalis* in the two groups. Seventy-nine percent of the FDR-RA were diagnosed with periodontitis and 15% of them had a severe form of the disease, while only 56% of the controls presented periodontitis. Obesity, ACPA and periodontitis were correlated to FDR-RA status. It was concluded that these three factors are relevant conditions associated with the development of RA in FDR-RA [67].

Similarly, Loutan et al. [68] evaluated the periodontal and rheumatological status of FDR-RA. ACPA positive (ACPA+) and ACPA negative (ACPA-) FDR-RA participants were included, in order to elucidate the correlation between periodontitis and seropositivity. Interestingly all ACPA+ subjects had periodontitis, with either a moderate (44.1%) or a severe form (47.1%) of the disease, while ACPA- participants, presented mostly mild (30.8%) and moderate (27%) periodontitis. In multivariable analyses, ACPA status and age were significantly and independently associated with periodontal conditions. The findings that periodontitis in FDR-RA is associated with ACPA seropositivity, suggest that periodontal disease precedes the development of RA in this population and acts as a trigger for RA [63, 68].

When focusing more on the periodontal pathogens, one study included 24 FDR-RA and 124 healthy individuals matched for age and sex. The prevalence of periodontitis in the FDR-RA group was similar to that of the control group (60.5% vs. 59%, respectively). The presence of P. *gingivalis* was more frequent in the FDR-RA (62.1%) compared to the control (42.7%) group and was associated to gingival inflammation and compromised periodontal status. However, anti- *Porphyromonas gingivalis* IgG1 and IgG2 antibodies were more frequent in controls than in the FDR-RA group [69].

Immune responses to P. *gingivalis* and their correlation with ACPA were investigated in a group of patients with RA and their FDR-RA. The study was performed to a unique cohort of North American Native people from central Canada who has one of the highest prevalence of RA globally. This population is also characterized by familial clusters of RA cases, early age of RA onset and a high prevalence of ACPA and RF. In this population, both RA patients and their FDR-RA presented anti-*Porphyromonas gingivalis* antibodies which were strongly associated with ACPA positivity. Their results indicate that immune responses to P. *gingivalis* affects the immune tolerance to citrullinated antigens which may lead to an increased risk of developing RA [70].

In another cross-sectional study, early RA patients, FDR-RA and healthy participants were included. Adipokine levels, clinical, joint radiological statuses and periodontal variables were assessed in order to evaluate if P. *gingivalis* could be a link between periodontitis and RA by decreasing the patient's immunological response. FDR-RA showed deteriorated periodontal status, obesity and high prevalence of ACPA. The authors concluded that obesity and periodontitis play a role in the development of RA in the FDR-RA group. Moreover, presence of P.*gingivalis* associates with the development of RA in this group [71].

Finally, Manoil et al. [72] examined the systemic responses against five periodontal pathogens in a cohort of four groups of FDR-RA divided according to the preclinical phases of RA. Serum IgG levels of the studied pathogens were not significantly different between the groups; they were associated neither with the preclinical phases of RA nor with ACPA seropositivity. However, significantly elevated serum levels of IgGs against the cluster of periodontopathogens and the red complex were found in all the ACPA-positive probands. These findings suggest that in individuals at risk of RA, periodontal bacteria as a complex, and not as single pathogens, contribute to the loss of immune tolerance to citrullinated antigens in terms of ACPA positivity.

## Discussion and Conclusions

RA is a considerable health problem that affects all areas in the life of the diseased patients. Early diagnosis and preventive measures may decrease the prevalence and severity of RA, and improve the quality of life of patients. Because of the association between RA and PD, especially in the early phases of the disease, PD should be assessed in the baseline assessment of patients at high risk. Health care practitioners should be aware of the association and active screening of at-risk individuals should be considered [73].

FDR-RA are at higher risk for developing RA, but only limited data exist for the role of PD. ACPA status seems significantly associated with deteriorated periodontal conditions and higher risk of RA. Factors, such as smoking, increased age and high BMI are associated with ACPA seropositivity and with deteriorated periodontal conditions, thus increasing the risk of developing RA.

P. *gingivalis* and has been identified as a possible trigger for RA via the breakdown of tolerance against citrullinated proteins and the formation of ACPAs. However, in the literature, contrasting evidence for its role in the etiopathogenesis of RA is found. A. *actinomycetemcomitans* is another potential candidate.

Larger studies evaluating all the potential mechanisms linking RA and periodontitis are needed. In particular, longitudinal studies evaluating the effect of PD on the risk of developing RA in FDR-RA are required to confirm the role of PD as a trigger for RA development. And last but not least, the effect of periodontal treatment on the development of RA needs to be established before preventive oral health interventions can be definitely established in at-risk populations for RA.

## Author Contributions

CG and AZ performed the literature research. All authors contributed to the article and approved the submitted version.

## Funding

AF's work is supported by research grant from the Swiss National Science Foundation (no. 310030E_205559/1; no. 320030_192471/1; no. 3200B0_120639).

## Conflict of Interest

The authors declare that the research was conducted in the absence of any commercial or financial relationships that could be construed as a potential conflict of interest.

## Publisher's Note

All claims expressed in this article are solely those of the authors and do not necessarily represent those of their affiliated organizations, or those of the publisher, the editors and the reviewers. Any product that may be evaluated in this article, or claim that may be made by its manufacturer, is not guaranteed or endorsed by the publisher.
